# Scientific Items

**Published:** 1888-12

**Authors:** 


### SCIENTIFIC ITEMS.

**The Anæsthetic Revelation.**—Within a few years it has been discovered that sulphuric ether often produces a most singular effect on the mind of the patient or experimenter who has inhaled it, giving rise to what has been called the "Anæsthetic Revelation." Just as the experimenter recovers from the anæsthetic, and before wide-awake consciousness fully returns, he has an intense perception of what seems to him at the time the philosphic secret of existence—the true explanation of the universe. This singular impression, though intense, does not last long, and in spite of the subject's strongest effort to carry the revelation out into wide-awake consciousness, he finds himself unable to do so, but is left full of awe by his strange experience, and wonder at the nearness of the solution which for so many ages has been sought so far a field. The present brief account has been gathered from the literature of the subject, which grows yearly. Mr. Benj. Paul Blood, of Amsterdam, N. Y., the discoverer of the phenomenon, originally made it known to psychologists in a pamphlet entitled *The Anæsthetic Revelation*, and he has since discussed its philosophical bearings in the *Journal of Speculative Philosophy*, Jan., 1886. The most scientific account appears in the *Therapeutic Gazette*, for August, 1886, where Dr. Geo. E. Shoemaker, of Philadelphia, relates his "Recollections After Ether-Inhalations." *Mind* discusses it in vol. iv, p. 345, and vol. vii, p 206. Dr. Oliver Wendell Homes mentions the experiment in his "Mechanism of Mind and Morals" (p. 46). A letter from the poet Tennyson concerning his own experience has recently reached the press; and valuable newspaper accounts of the phenomenon may be found in the *Tribune*, September 3, 1886; the *Evening Post*, October 30, 1886, and the *Utica Herald*, September, 15, 1886.

The abstract, philosophic nature of the ether dream (says a writer in *The Open Court*) gives it a special interest to students of philosophy and pyschology. By its intensely specific character it differs entirely from the opium or hashish hallucination. The opium-eater may dream of a thousand different things; but the ether patient invariably has one fixed impression, a belief that the ultimate secret and explanation of existence stands revealed to him as finite knowledge never has and never could reveal it. The singular thing is that this impression may happen to a man who has never given one thought to philosophy, and whose mind therefore is devoid of materials for this impression. This fact, and the specific likeness of the effect of the ether on all who have made the experiment, has led some psychologists to declare the impossibility of considering the phenomenon a dream, and to claim place for it as genuine philosphic insight. Further and more accurate experimenting will throw clearer light on what is one of the most remarkable discoveries known to modern pscychology. —*N. Y. Medical Times*.

**Causes of Fire.**—In regard to spontaneous combustion, the fires of the year in Boston have furnished some new observations of considerable importance. In one case, says the *American Architect*, a quantity of feather dust in a bedding manufactory took fire without apparent reason. It was found, however, that a piece of thick glass had been lying on the ferthers, and the sun's rays, concentrated in some way by the glass, had set fire to them, although the day was a cold one in the month of March. In another case, a number of tarpaulin hats were lying, packed together, in a window. The high temperature, with, perhaps, the c ose packing of the hats, caused them to burst into a blaze. Two other fires were caused by putting paraffne paper, such as candy is wrapped in, into a refuse barrel which contained a little sawdust; and a third, which destroyed twenty thousand dollars worth of property, was occasioned by putting greasy paper, which had been used to wrap lunches in, into a wooden refuse barrel, which contained some sawdust and sweepings.—*Science.*

**One** of the interesting features of the approaching French exhibition is to be a model of the earth, made on the scale of one-millionth. It is to be accurately constructed, and will rotate on its axis. Even on this scale it will be an enormous object, nearly 21 feet in diameter.—*Scientific American.*

**A Toad in Solid Coal.**—The correspondent of the *Colliery Guardian* reports a case which, if true, must be interesting to geologists. In the Coleford district of the Forest of Dean a small colliery has been opened, and while a collier was engaged in breaking up a fall of block coal, he found a toad in the centre. It seemed firmly embedded in the coal, and it was alive. Its form was imprinted upon the face of the mineral, and the animal is still living The incident has occasioned much interest in the neighborhood.—*Scientific American.*

**A Correspondent** of the *Army and Navy Journal* asks: "What is the longest piece of ordnance thas has ever been successfully fired ?" and receives the following answer: "If you include in the term ordnance everything that carries a projectile, we should answer fourteen miles. This is the straight tube conveying natural gas from Murrayville to Pittsburg. To clear this out, a projectile known as the 'gum ball' wes inserted in the end at the gas well, closely fitting the interior. The gas was then turned on full force and the gum ball fired its full length, coming out the further end in a few minutes."—*Scientific American.*

**The Longest Tangent in the World.**—The new Argentine Pacific Railroad from Buenos Ayres to the foot of Andes, has on it what is proably the longest tangent in the world. This is 340 kilometers (211 miles) without a curve. In this distance there is not a single bridge and no opening larger than an ordinary culvert, no cut greater than one meter in depth, and no fill of a height exceeding one meter. There is almost an entire absence of wood on the plain across which the western end of the road is located. This has led to the extensive use of metallic ties, which will be employed on nearly the entire road —*Scientific American.*

